# Isotope partitioning between cow milk and farm water: A tool for verification of milk provenance

**DOI:** 10.1002/rcm.9160

**Published:** 2021-09-27

**Authors:** Marta Boito, Paola Iacumin, Mattia Rossi, Nives Ogrinc, Giampiero Venturelli

**Affiliations:** ^1^ Department of Chemistry, Life Sciences, and Environmental Sustainability, Parco area delle Scienze 157/A University of Parma Parma Italy; ^2^ Department of Environmental Sciences Jožef Stefan Institute Ljubljana Slovenia

## Abstract

**Rationale:**

The oxygen and hydrogen isotope compositions of the water component of the milk from nine Italian dairy farms were studied together with the farm water for one year. The aim was to verify the importance of farm water and seasonal temperature variation on milk isotope values and propose mathematical relations as new tools to identify the milk origin.

**Methods:**

Milk was centrifuged to separate the solids and then distilled under vacuum to separate water. δ(^18^O/^16^O) and δ(^2^H/^1^H) analyses of the water molecules were carried out using a water equilibrator online with a mass spectrometer. For oxygen and hydrogen isotope determination, water was equilibrated with pure CO_2_ for 7.5 h and with pure H_2_ for 5 h, respectively. The isotope ratio value is indicated with δ (expressed on the VSMOW/SLAP scale) as defined by IUPAC.

**Results:**

The average annual isotope value of milk at the different cattle sheds is mostly related to the farm water suggesting that the drinking water is the most important factor influencing the isotopic values of the milk water. The milk/water fractionation factor correlates with the milking time and, thus, the seasonal temperature is best described by a 4^th^ order polynomial regression line. A two‐level check model was used to verify the milking provenance.

**Conclusions:**

This study shows that it is essential to analyze both milk and farm water to indicate provenance. A two‐step verification tool, based on the difference between the measured and calculated δ(^18^O/^16^O)_M_ values, and the difference between the calculated and estimated milk‐water fractionation factors, allowed the source determination of milk. Both conditions must be met if the milk is considered to be from the Parmigiano‐Reggiano production region. Although this approach was developed for this region, it can easily be tested and adapted to other dairy production areas.

## 1 INTRODUCTION

Several empirical studies have demonstrated the utility of oxygen and hydrogen stable isotope measurements on milk for verifying its origin.^1^, ^2^, ^3^, ^4^, ^5^, ^6^, ^7^, ^8^, ^9^, ^10^, ^11^, ^12^, ^13^ In fact, like many other natural products, milk retains its isotopic features acquired at the time of its production. The δ(^18^O/^16^O) and δ(^2^H/^1^H) values (symbols used according to IUPAC – International Union of Pure and Applied Chemistry)^14^ of milk depend on (i) the main sources of oxygen and hydrogen and (ii) on the animal's metabolism, both of which are influenced by climatic conditions. The sources of H and O in an animal's body water and milk water are drinking water, food and air.^3^ In most cases, drinking water is taken from local groundwater (GW) sources and thus correlates with the geo‐climatic characterization of the area of origin. Only a few studies have directly investigated the relationship between the isotopic composition of milk water and drinking water (hereby indicated as farm water).^9^, ^10^, ^11^, ^12^, ^13^ All these studies indicate that the δ(^18^O/^16^O) and δ(^2^H/^1^H) values in milk water were isotopically enriched with the heavier isotope relative to cow drinking water. For δ(^18^O/^16^O), an enrichment of 2–6‰ was documented.^6^, ^9^, ^10^, ^11^, ^12^ Seasonal variations in the H and O isotopic compositions in milk water have also been observed, with higher values on summer days and lower values during winter. A relationship between δ(^18^O/^16^O) in milk water and the season was reported by Kornexl et al^10^ and Rossman.^11^ The authors found that seasonal changes in the δ(^18^O/^16^O) of forage plants and an animals' body are linked to evapotranspiration. A significant relationship between the isotopic composition of milk and both farm water and feed was also demonstrated by the study performed by Ehtesham et al^9^ in New Zealand. As far as animal metabolism is concerned, Ritz et al^15^ and Abeni et al^7^ report that the δ(^18^O/^16^O) value of animal water present in the different body fluids (urine, milk, plasma) is not influenced solely by drinking water. For example, Midwood et al^16^ showed that, in ruminants, methane production results in ^2^H enrichment of body water due to depletion in methane. The δ(^2^H/^1^H) and δ(^18^O/^16^O) values in milk were also used to verify geographical origin based on the relationship between the isotopic signature of milk and the drinking water of regions at different latitudes and/or altitudes.^4^, ^13^ As an application example, in the study performed by Chesson et al,^13^ δ(^18^O/^16^O) values in milk water were used to predict possible regions of origin for restaurant samples.

The verification of geographical origin is essential for particular local foodstuffs with Protected Designation of Origin (PDO) to protect their authenticity. The PDO designation of Parmigiano‐Reggiano, the most famous region of cheese production, means that the milk used in its production must come from a defined region in the Po River plain and the northern side of the Apennine chain (Northern Italy). However, the remarkable price differences among cheeses makes it tempting for criminals to use fraudulent designation labels on products that do not correspond to the authentic production areas. In order to protect the consumer and assure honest competition on the market, it is desirable to develop objective and robust methods to verify the authenticity and origin of economically important products such as Parmigiano‐Reggiano cheese. This paper investigates the isotopic signature of cow milk from this region concerning (i) farm water during the different periods of the year and (ii) local temperature, which influences evapotranspiration and animal's metabolism. The work describes a model for verifying the isotopic composition of milk characteristic of the area of production of the Parmigiano‐Reggiano cheese.

## 2 MATERIALS AND METHODS

### 2.1 Selection of cattle sheds

**FIGURE 1:**
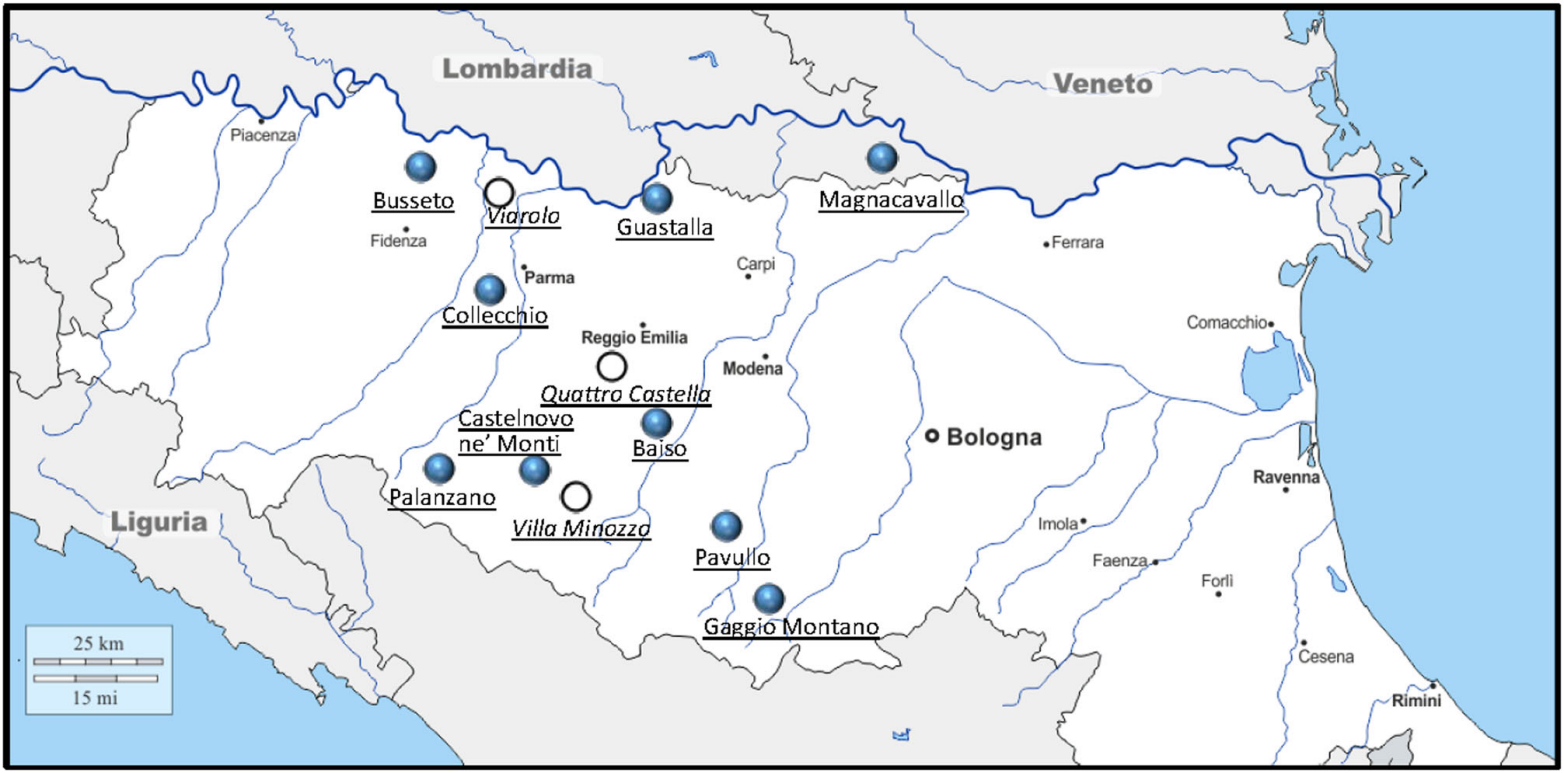
Location of the investigated cattle sheds (filled circles). The open circles refer to cattle sheds used for validation of the model reported in the text

Nine cattle sheds were randomly selected among the cattle sheds from the region of the Parmigiano‐Reggiano cheese production. These sheds are located at Collecchio (Parma province), Busseto (Parma), Castelnovo ne’ Monti (Reggio Emilia), Guastalla (Reggio Emilia), Baiso (Reggio Emilia), Palanzano (Parma), Gaggio Montano (Bologna), Pavullo (Modena), and Magnacavallo (Mantova) (Figure 1). In addition, four sheds – Torrile, Villa Minozzo, Quattro Castella, and Viarolo – were also sampled for model verification.

### 2.2 Samples

Hydrogen and oxygen isotope determinations were carried out monthly from February 2018 to January 2019 on 108 samples of farm water and 108 samples of cow milk (average daily milk). Samples of farm water (100 cm^3^ in double‐cap containers) were stored at 2°C and milk (50 mL in double‐cap containers) at −20°C to avoid bacteria proliferation. The number of cattle per farm (≥40) is such that any influence on the milk isotopic composition linked to an individual animal (breed, time since to the last pregnancy, age, etc.) is taken into account. Milk was centrifuged (4000 rpm for 4 min) to separate the solids and distilled under vacuum to separate the water.

### 2.3 Stable isotope analysis

The δ(^18^O/^16^O) and δ(^2^H/^1^H) analyses were performed at the Isotope Geochemistry Laboratory of the University of Parma (Italy) using a water equilibrator (HDO device, Thermo‐Finnigan, at 18°C) online with a Finnigan Delta XP mass spectrometer. For oxygen isotope determination, 5 cm^3^ of water was equilibrated with pure CO_2_ for 7.5 h, while for hydrogen isotopes, 5 cm^3^ of water was equilibrated with pure H_2_ for 5 h (platinum wire was used as a catalyzer of gas–liquid equilibration). The isotope ratio value is expressed using δ notation (VSMOW/SLAP scale) defined by IUPAC (International Union of Pure and Applied Chemistry). In the case of oxygen:

δO18/O16sp/VSMOW=RO18/O16spRO18/O16VSMOW–1=RO18/O16spRO18/O16VSMOW–1103‰

where sp = farm water (W) or milk water (M), R(^18^O/^16^O) = ratio of the isotopic abundances of O18 and O16, ‰ = 10^−3^, VSMOW = primary international standard of reference. A similar equation may be written for the hydrogen isotopes H2 and H1. The standard prediction uncertainties^17^, ^18^ for measurements of the samples were ±0.08‰ for δ(^18^O/^16^O)_W_ and ±1‰ for δ(^2^H/^1^H)_W_ in farm water, and ±0.15‰ for δ(^18^O/^16^O)_M_ and ±1.5‰ for δ(^2^H/^1^H)_M_ in milk water.

### 2.4 Calculations and statistical analysis

The isotope fractionation between milk water (M) and farm water (W) was calculated using the following relationship:

(1) $$\alpha_{t}=\frac{\delta_{M,t}+1\;}{\delta_{W,t}+1}$$

where $\alpha_{t}$ is the isotope fractionation factor at the time *t*, while $\delta_{W,t}$ and $\delta_{M,t}$ are δ(^18^O/^16^O) or δ(^2^H/^1^H) values in water and milk at time *t*.

Trends in δ(^18^O/^16^O)_M_ and $\alpha_{t}$ values were plotted against time. The distribution of the points was smoothed according to the LOESS smoothing procedure (algorithm LOWESS – Locally Weighted Scatterplot Smoothing^19^, ^20^). LOESS (smoothing parameter = 0.2) nicely describes the data distribution, but it does not generate a mathematical formula. In our case, the smoothing lines are very similar to the 4^th^ order polynomial regression lines:

(2) 103δO18/O16Morαt=At4+Bt3+Ct2+Dt+E

where A, B, C, D, and E are regression coefficients determined by fitting the curve to experimental data.

## 3 RESULTS

Hydrogen and oxygen isotope data for the nine cattle sheds investigated are presented in Table S1 (supporting information).

### 3.1 Oxygen and hydrogen isotopes in farm water (W)

The δ(^18^O/^16^O)_W_ and δ(^2^H/^1^H)_W_ values for the farm waters are in the range −11.39‰ to −7.83‰ and −80.7‰ to −50.1‰, respectively. Assuming, as a first approximation, that the standard deviation is representative of the dispersion of the data, except for the cattle sheds of Pavullo, Gaggio, and Baiso, the standard deviation in the δ(^2^H/^1^H)_W_ values (0.4–1.3‰) (Table S1, supporting information) is similar to the prediction uncertainty. Moreover, except for the Pavullo cattle shed, standard deviations of δ(^18^O/^16^O)_W_ (0.05–0.13‰) are close to the prediction uncertainty. Thus, the isotope ratio of farm waters does not change significantly during the year, but, when it does, it is due to the contribution of different water sources during the year. This variation is particularly evident at Pavullo, where the δ(^2^H/^1^H)_W_ and δ(^18^O/^16^O)_W_ values of the 12 monthly samples are well correlated (R = 0.98).

Farm waters from the cattle shed of Guastalla have δ(^18^O/^16^O)_W_ = −9.40 ± 0.05‰, which could indicate a mixed Alpine‐Apennine origin.

**FIGURE 2:**
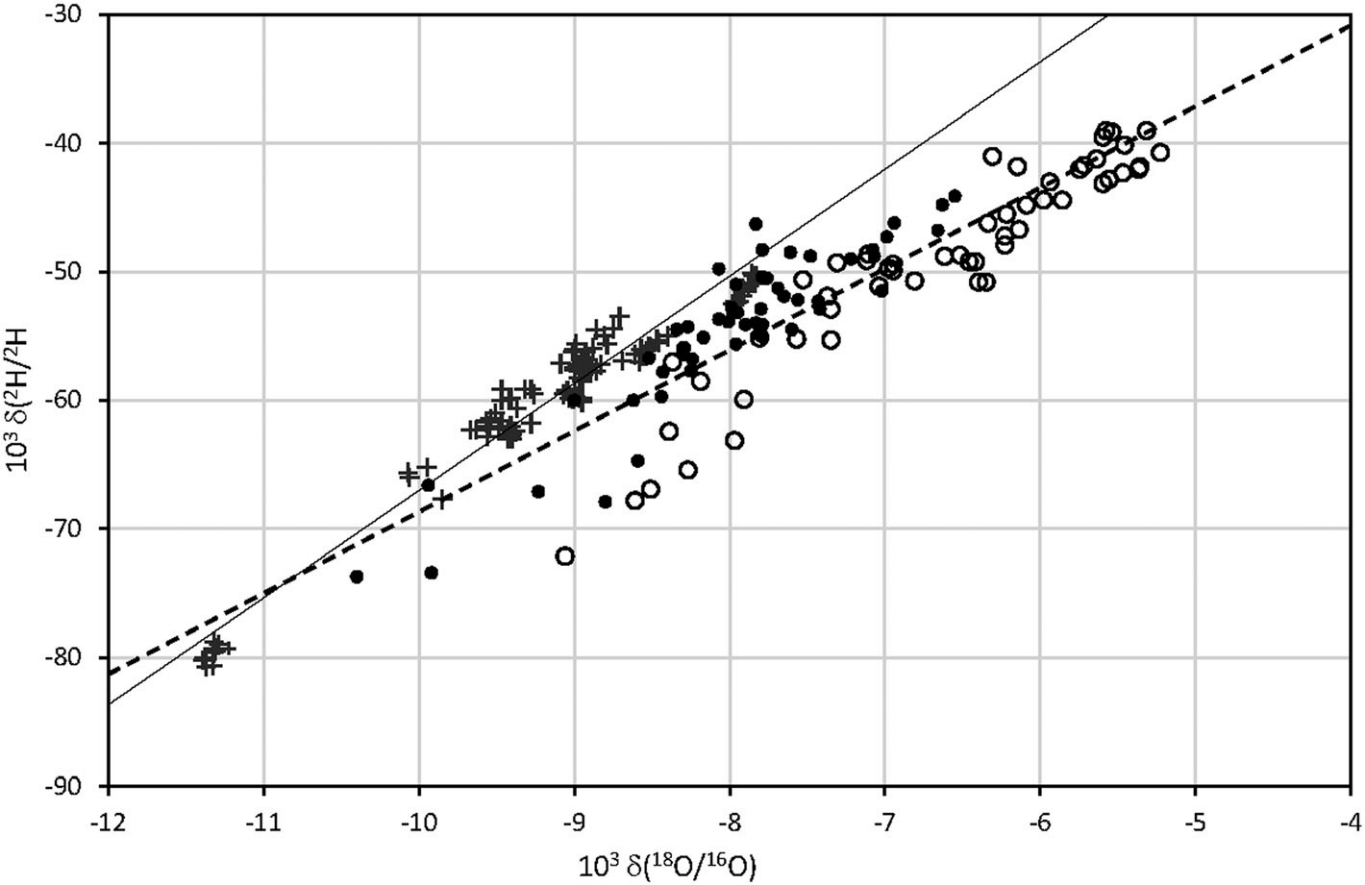
Diagram of regressions of δ(^2^H/^1^H) on δ(^18^O/^16^O) for farm and milk water. Crosses = farm water; filled circles = milk water for November, December, January, February, March, April; open circles = milk water for May, June, July, August, September, October (data are referred to VSMOW)

Figure 2 shows the regressions δ(^2^H/^1^H) on δ(^18^O/^16^O) for the farm waters (W). The regression line 1 is as follows:

(3) δH2/1HW=8.33±0.17δO18/16OW+16.3±1.6‰

where R^2^ = 0.96 and s(yx) = 1.6‰.

R^2^ is the determination coefficient and s(yx) the standard error of the regression.

### 3.2 Oxygen and hydrogen isotopes in milk water (M)

The δ(^18^O/^16^O)_M_ and δ(^2^H/^1^H)_M_ values for milk range from −10.40‰ to −5.23‰ and −73.7‰ to −39.0‰, respectively, and the standard deviation is, in most cases, greater than the prediction uncertainty.

For milk water (M) the following regression line 2 was obtained (Figure 2):

(4) δH2/1HM=6.30±0.28δO18/16OM−5.6±2.1‰,

where R^2^ = 0.83 and s(yx) = 3.2‰.

Despite the fact that the isotope ratios of most of the single farm waters do not change during the year, milk waters are particularly enriched in O18 during summer and generate an overall δ(^2^H/^1^H)_M_ and δ(^18^O/^16^O)_M_ slope that is lower than the slopes obtained for farm water (Figure 2). During summer, heavy isotope enrichment is particularly evident at Castelnovo and Baiso, where the data for milk exhibit bimodality for hydrogen and oxygen. Moreover, for all the cattle sheds as a whole (Figure 2) and each cattle shed considered separately, the slope of each line δ(^2^H/^1^H)_M_ on δ(^18^O/^16^O)_M_ is lower than that for the farm waters. In addition, the obtained slopes are negatively correlated with the average local annual temperature (R = 0.71), which supports an animal response to the temperature variation similar to that produced by evaporation processes. We come to the same conclusion considering the so‐called “deuterium excess” defined as d_ex_ = 10^3^ [δ(^2^H/^1^H) − 8 δ(^18^O/^16^O)], a value that decreases as evaporation increases. The difference between the deuterium excess for the farm water (W) and the milk water (M), d_ex_,_W_ − d_ex_,_M_, increases with increasing local temperature. The best correlation is obtained considering the average temperature for the 2 weeks preceding the date of sampling (R = 0.80) since cows take at least 14 days to equilibrate internal fluids with the ingested water.

**FIGURE 3:**
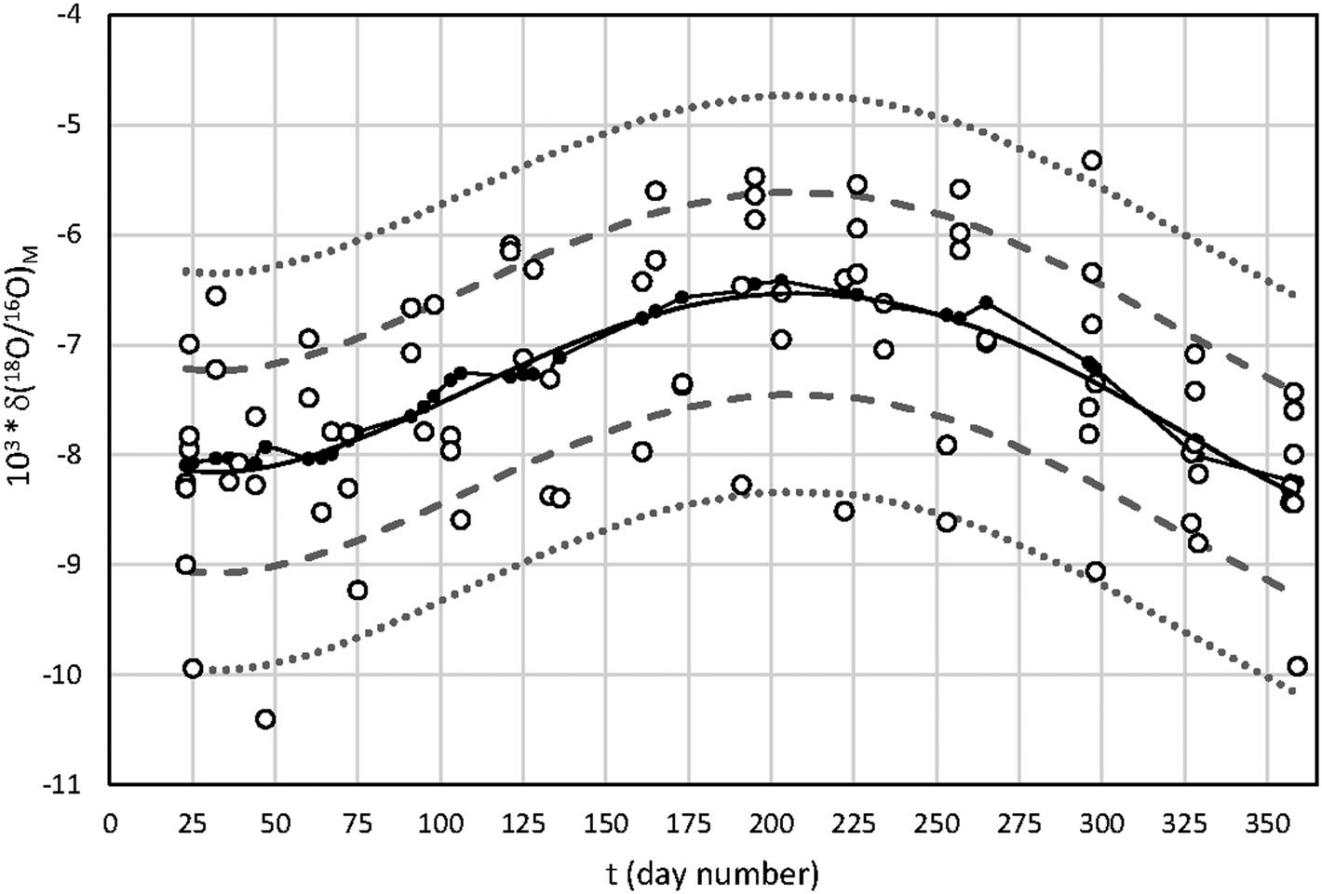
10^3^ δ(^18^O^/16^O)_M_ values vs time (*t*) for milk water (Castelnovo and Baiso excluded). Continuous line with black circle = LOESS smoothing line (smoothing factor = 0.2); continuous line = 4th order polynomial regression 10^3^ δ(^18^O/^16^O)_M_ = 1.55*10^−9^ *t* ^4^ *–*1.34*10^*−6*^ *t* ^3^ + 3.24*10^−4^ *t* ^2^–1.62*10^−2^ *t* – 7.91, n = 84, R^2^ = 0.30, s(yx) = 0.92‰ (see text); discontinuous lines include the band delimitated by $\hat{Y}$ – s(yx) and $\hat{Y}$ + s(yx); dotted lines include the band delimitated by $\hat{Y}$ – 1.645 s(yx) and $\hat{Y}$ + 1.645 s(yx)

The change in the isotopic values of the milk water during the year is shown in Figure 3, where δ(^18^O/^16^O)_M_ is reported against *t*, the number of yearly days calculated starting from January 1st of a non‐leap year. The 4th order polynomial regression lines (Equation 2) were used to smooth the obtained data. The equation is reported (Figure 3) without taking into account Castelnovo and Baiso:

(5) 103δO18/O16M=1.55*10−9t4–1.34*10−6t3+3.24*10−4t2–1.62*10−2t–7.91

where the number of data used in the polynomial regression (n) = 84, coefficient of determination (R^2^) = 0.30, and the standard error of the regression (s (yx)) = 0.92‰. This equation will be used later.

## 4 DISCUSSION

### 4.1 Distribution of isotopes between farm water and milk water

Water consumed by cows kept indoors is either groundwater from the cattle shed wells or potable water from the local water supply. In the Po plain area, only a small amount of water derives from local precipitation; groundwater mostly comes from the Apennine and, more rarely, from Alpine chains.^20^, ^21^ The cattle shed at Magnacavallo (Mantova province), close to the Po River, gives an annual average δ(^18^O/^16^O)_W_ = −11.33 ± 0.05‰ (Table S1, supporting information); this constancy over time excludes any mixing with recent precipitation and indicates an old water origin. Gorgoni et al^21^ and, more recently, Martinelli et al^22^ identified groundwaters with δ(^18^O/^16^O)_W_ values less than about −10.5‰, whereas today the δ(^18^O/^16^O) of the Po River is around −9.5‰. These waters are considered to be of Alpine origin and infiltrated more than 9000 years ago.

The farm water line (line 1; Figure 2) and milk water line (line 2; Figure 2) differ significantly in terms of slope and intercept. Starting from any point on the water line, water evaporation should result in a straight line with a lower slope and intercept,^23^ which is observed for the milk water line. On the other hand, with only few exceptions, no significant water evaporation of the drinking water occurs. Thus, for milk water a trend is obtained that simulates evaporation processes. A similar trend (δ(^2^H/^1^H)_M_ = 5.15 δ(^18^O/^16^O)_M_ − 7.24‰; R^2^ = 0.54) has been reported by Ehtesham et al^9^ for milk from New Zealand dairy farms.

The δ(^18^O/^16^O)_M_ vs δ(^18^O/^16^O)_W_ and δ(^2^H/^1^H)_M_ vs δ(^2^H/^1^H)_W_ values are significantly correlated (probability $p_{\mathrm{slope}=0}$ << 0.00010 for both the regression lines) and the determination coefficient R^2^ = 0.41 and R^2^ = 0.69, respectively. The R^2^ values indicate that about 41% of the variance in the δ(^18^O/^16^O)_M_ values and 69% of the variance in the δ(^2^H/^1^H)_M_ values are explained by δ(^18^O/^16^O)_W_ and δ(^2^H/^1^H)_W_ values of the farm water, respectively. When the annual average values for each cattle shed are considered, the determination coefficient is very high (R^2^ = 0.93) for both oxygen and hydrogen, suggesting that the annual average values of δ(^18^O/^16^O)_M_ and δ(^2^H/^1^H)_M_ for the different cattle sheds are explained by the δ(^18^O/^16^O)_W_ and δ(^2^H/^1^H)_W_ values of farm waters. The milk water is generally enriched in the heavier isotopes of H and O compared with milk water. Ehtesham et al^9^ report an oxygen enrichment of approximately 4‰ for milk water compared with farm water and Kornexl et al^10^ and Rossman^11^ an ^18^O enrichment of 2–6‰ compared with groundwater and other water sources such as grass, silage and hay (water represent up to 85% of grass, 75% of silage and 15% of hay). Our study indicates an overall ^18^O enrichment ranging from 0.5 to 3.7‰ in milk water. In the case of hydrogen, the ^2^H enrichment is much higher reaching up to 18‰. These values are comparable with those reported by Chesson et al,^13^ where the average difference between milk and cow drinking water was 11‰ for δ(^2^H/^1^H) values and 2.2‰ for δ(^18^O/^16^O).

**FIGURE 4:**
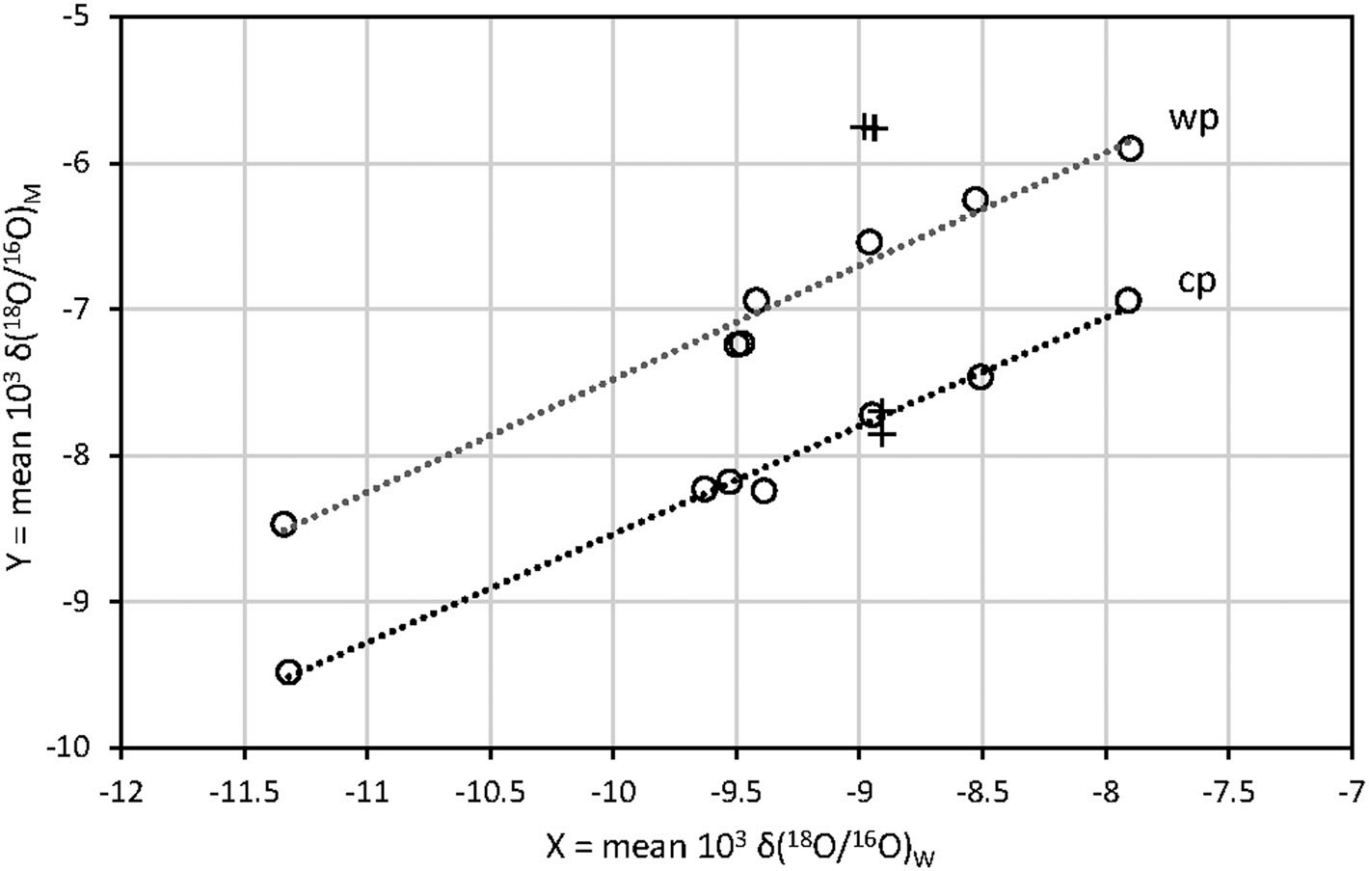
Relationship between average 10^3^ δ(^18^O/^16^O)_M_ values for milk water and 10^3^ δ(^18^O/^16^O)_W_ values for farm water at the different cattle sheds for the “warm period” (wp) and the “cold period” (cp) (Castelnovo and Baiso excluded). Regressions: Y_wp_ = 0.774 (±0.049) X_wp_ + 0.27 (±0.46), R = 0.996; Y_cp_ = 0.774 (±0.049) X_cp_ − 1.14 (±0.28), R = 0.990; p_same slope_ = 0.56. Cross: Castelnovo and Baiso, where, during summer, the animals are kept outdoors (data referred to VSMOW)

The δ(^18^O/^16^O) and δ(^2^H/^1^H) values for milk may change significantly depending on whether the animals (cows) are kept outdoors during the warm season. For instance, Abeni et al^7^ report a difference of about 1‰ for oxygen and 15‰ for hydrogen between summer and winter, while Gregorčič et al^6^ report a difference up to 4.5‰ for oxygen. We also compared the isotopic values (Figure 4) for the “cold period” (November, December, January, February, March, April) and “warm period” (May, June, July, August, September, October). The warm period trend exhibits two data points above the main trend, i.e., the δ values are higher than expected by about 0.90–0.94‰. These points refer to Castelnovo and Baiso, where, during summer, the animals are kept outdoors. If these two points are not considered, the regression lines for the two periods of the year do not exhibit a significant difference in the slopes (p_same slope_ = 0.56). This result is because when outdoors, evapotranspiration is greater, and the animals may drink evaporated water and eat grass with an elevated isotope ratio due to evapotranspiration.^24^, ^25^, ^26^ The two regression lines for Castelnovo and Baiso indicate that the animals' biological responses during the year are similar for all the investigated cattle sheds. Comparable results are obtained using hydrogen isotopes.

### 4.2 The isotopic fractionation factor between milk water and farm water and its dependence on the sampling time

The isotopic fractionation factor between milk water and farm water has been further evaluated using Equation 1. Consider the $\delta_{W},t$, value for water sampled at a generic cattle shed at a generic time *t*; *t* is the number of days calculated starting from January 1st of a non‐leap year, and the $\delta_{M},t$, value for the milk sampled at the same cattle shed and at the same time *t* as for $\delta_{W},t$.

The important role that sampling time has on $\alpha_{t}$ is demonstrated and quantified by the two‐way ANOVA (ANalysis Of VAriance). Assuming as a first approximation that for all the cattle sheds monthly sampling was performed at the same time $\bar{t}$, two‐way ANOVA – where the columns are referred to $\alpha_{t}$ for the cattle shed *i* and the row to the time $\bar{t}$ of sampling – indicates that most of the variance (s^2^) associated with the $\alpha_{t}$ values is related to the sampling time $\bar{t}$. This association is particularly evident for oxygen: actually, $s_{\bar{t}\left(\mathrm{row}\right)}^{2}$ = 5.23*10^−6^ is largely higher than $s_{i\left(\text{column}\right)}^{2}$ = 0.98*10^−6^; in addition, $s_{\text{error}}^{2}$ = 0.21*10^−6^ includes analytical error and other fluctuations due to an animal's metabolism. Therefore, the monthly isotopic values of milk water are influenced by sampling time, and the cattle shed investigated.

**FIGURE 5:**
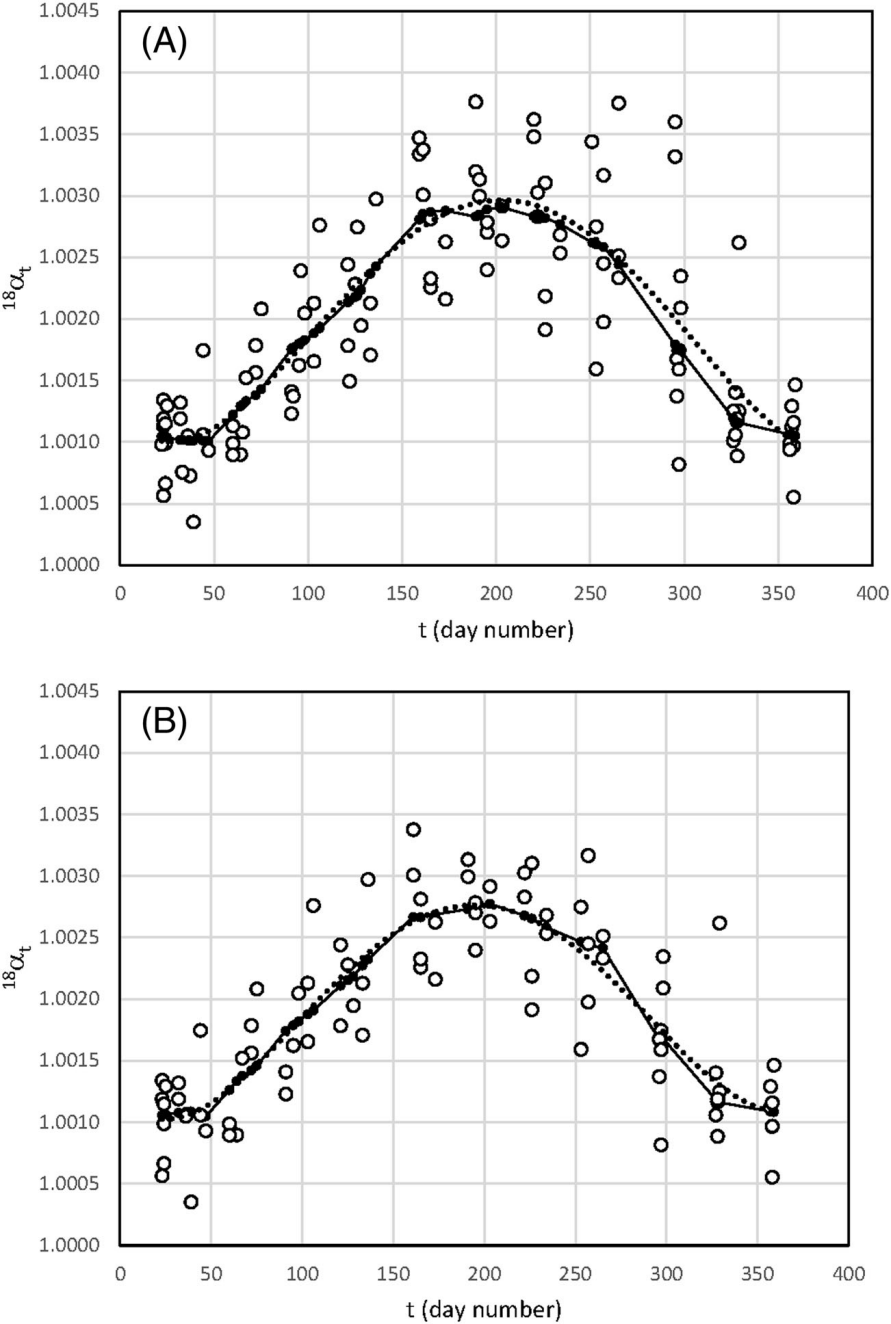
α18t values vs time (*t*) for (A) all the data and (B) Castelnovo and Baiso excluded. The continuous lines represent the trends obtained using LOESS smoothing (smoothing constant = 0.2), the dotted lines are 4th order polynomial regression lines used in the text

The distribution of the data points in the $\alpha_{t}$ vs *t* plot (Figure 5) may be smoothed according to LOESS. This smoothing is done considering all the data (Figure 5A) and disregards Baiso and Castelnovo (Figure 5B). The generated smoothing lines are very similar to 4th order polynomial regression lines, as shown by the regression of oxygen isotopes (Equation 5). Only equations related to oxygen isotopes are discussed, but lines may be obtained for hydrogen isotopes as well.

#### 4.2.1 All the sheds

(6a) α^t18=2.072*10−12t4–1.689*10−9t3+3.867*10−7t2−1.770*10−5t+1.0012

where the number of samples n = 108, s (yx) = 0.50‰, and R^2^ = 0.69. Because of the complexity of this equation, it is not possible to calculate correctly the standard prediction uncertainty, $u\left(\hat{\alpha }\right)$, on α^18t for a sample related to a new cattle shed. Approximately, the uncertainty was calculated according to the relationship:

$$u\left(\hat{\alpha }\right)\approx t_{\alpha \left(2\right),\nu }\;\sqrt{s{\left(\mathrm{yx}\right)}^{2}+\frac{s{\left(\mathrm{yx}\right)}^{2}\;}{n}\;}$$

However, this underestimates the $u\left(\hat{\alpha }\right)$ value. In our case, $u\left(\hat{\alpha }\right)\;$≈ 0.51‰.

#### 4.2.2 Excluding Castelnovo and Baiso

(6b) α^t18=2.096*10−12t4–1.627*10−9t3+3.540*10−7t2−1.467*10−5t+1.0012

where n = 84, s (yx) = 0.41‰, R^2^ = 0.72, and $u\left(\hat{\alpha }\right)\;$≈ 0.42‰.

The error of the regression is typically lower for the line obtained from Equation 6b than for the line calculated with Equation 5 since for milk water the α^t18 value is normalized to that of farm water.

Since the fractionation factor, $\alpha_{t}$, and temperature change during the year, it is reasonable to correlate $\alpha_{t}$ to temperature. Here, the temperature ${\bar{T}}_{i}$ is considered the average temperature during the 2 weeks preceding the monthly sampling for each generic locality *i* (Table S1, supporting information). This means that for each cattle shed *i* a regression line ^18^ α^i,t = B_i_ ${\bar{T}}_{i}$ + A_i_ can be defined. From the results (Table 1), it is clear that α^i,t18 and ${\bar{T}}_{i}$ are always strongly correlated (R = 0.82–0.94), and the large variation in δ(^18^O/^16^O)_M_ values during the year is related to the change in temperature through its influence on an animal's metabolic response. As expected, Castelnovo and Baiso exhibit the highest slope (B_i_ of 13.3 and 11.8, respectively) because during the “warm period” animals are kept outdoors and the $\alpha_{t}$ value significantly increases.^27^ These results also agree with those of Abeni et al^7^ who found that, during summer, the hydrogen and oxygen isotope ratios in the body fluids of cows increase drastically.

**TABLE 1:** Coefficient of correlation, R, and slope, B_i_, for the linear regression α18i,t = B_i_ ${\bar{T}}_{i}$ (°C) + A_i_, where $\bar{T}\left({}^{\circ}C\right)$ is the local average temperature for the 2 weeks preceding the date of sampling (single data for $\bar{T}$ are reported in Table S1, supporting information)

Localitiy	R	10^5^ B_i_
Collecchio	0.94	6.90
Guastalla	0.91	7.72
Gaggio Montano	0.84	6.29
Busseto	0.92	5.72
Baiso	0.91	11.8
Pavullo	0.82	7.03
Castelnovo ne’ Monti	0.79	13.3
Palanzano	0.82	8.97
Magnacavallo	0.85	7.39

Note that the highest values of B_i_ are for Baiso and Castelnovo, where, during summer, the animals are kept outdoors.

The heavy isotope enrichment of the milk is related to the isotope fractionation during water evaporation and CO_2_ production. During the summer, a dairy cow reacts to the high temperature by restoring its metabolism; the symptomatology will be characterized by an increase in body temperature by half a degree centigrade and an increased respiratory rate of over 80 beats per minute, directly affecting CO_2_ production. This response increases the metabolic water production and, as this water is exhaled through the lungs, enhances the oxygen isotope fractionation. During this attempt to acclimatize and in full heat stress, a cow will reduce its milk production; a different production rate could directly modify the fractionation of oxygen in the udder between body and milk water.^10^, ^28^

### 4.3 Two‐step check to verify milk origin

A two‐step check to verify milk origin is proposed based on the fractionation factor between milk and water. For animals living indoors, it is important to verify the model's validity for other cattle sheds not used to construct the model but belonging to the same region of Parmigiano‐Reggiano cheese production. For this purpose, four new sheds located at Torrile, Viarolo, Villa Minozzo, and Quattro Castella were selected (Table 2a).Step one: first, the milking at time *t* was verified using the following condition:

(7) δO18/O16M,m,t−δO18/O16M,c,t≤1.645*syx5

where δO18/16OM,m,t is the measured value, δO18/16OM,c,t is the corresponding value calculated with Equation 5, s(yx)_(5)_ the standard error of the regression (Equation 5; the band defined by $Y_{5}$ = δ(O18/O16)M,c,t ± 1.645 s(yx)_(5)_ includes about 90% of the population data around the line (5) (Figure 3). Since the measured and calculated milk isotope values for the new cattle sheds agree with Equation 5, at the first level of investigation, the milk is compatible with the investigated area (Table 2a).

**TABLE 2:** (a) Data for new cattle sheds *belonging* to the area of production of the Parmigiano‐Reggiano cheese; (b) data for cattle sheds that are *declared* by the farmers as belonging to the area of production of the Parmigiano‐Reggiano cheese marked L1, L2, L3, and L4. The two‐step criteria are also included

Locality of milk provenance	Date of sampling (day/month/year)	*t* (day number)	10^3^ δ(^18^O/^16^O)_W,m,t_	10^3^ δ(^18^O/^16^O)_M,m,t_	10^3^ δ(^18^O/^16^O)_M,c,t_ (Equation 5)	10^3^ Δ < 1.51	^18^ *α*_m,t_	^18^ *â*_t_ (Equation 6b)	10^3^ Δ < 0.45
a)									
Torrile	20/01/2019	20	−8.78	−8.16	−8.11	0.05	1.000625	1.001036	0.41
Torrile	25/02/2019	56	−8.86	−7.55	−8.02	0.47	1.001322	1.001224	0.10
Villa Minozzo	05/02/2019	36	−9.10	−7.81	−8.13	0.32	1.001302	1.001058	0.24
Quattro Castella	05/02/2019	36	−8.77	−7.92	−8.13	0.21	1.000858	1.001058	0.20
Viarolo	24/12/2018	222	−7.51	−6.75	−8.21	1.46	1.000766	1.001096	0.33
b)									
L1	08/08/2019	220	−9.83	−6.90	−6.43	0.47	1.002959	1.0026919	0.27
L2	01/03/2019	150	−8.83	−7.26	−6.79	0.47	1.001584	1.002534	***0.95***
L3	01/07/2019	182	−8.15	−6.73	−6.50	0.23	1.001432	1.002747	***1.32***
L4	02/02/2021	30	−9.32	−7.20	−8.14	0.94	1.002140	1.001036	***1.10***

*t*, day number starting from January 1st of each non‐leap year;

δ(^18^Ο/^16^Ο)_W,m,t_, δ(^18^Ο/^16^Ο)_M,m,t_ = values measured for farm water and milk water, respectively;

10^3^ Δ = 10^3^|δ(^18^O/^16^O)_M,m,t_ ‐ δ(^18^O/^16^O)_M,c,t_|, absolute value: Δ ≤ 1.645 s (yx)_(3)_) = 1.51‰, s (yx)_(3)_ = standard error of regression (5) = 0.92‰; 1.645 = coverage factor at significance level 0.10 (Figure 3);

^18^ *α*_m,t_ = value calculated from δ(^18^Ο/^16^Ο)_W,m,t_ and δ(^18^Ο/^16^Ο)_M,m,t_ at time the *t*;

α^t18 values estimated according to Equations 6b; Δ = |^18^ *α*_m,t_ ‐ α^t18 │, absolute value: Δ ≤ 0.45‰ (see text).

Step two: second, to check that the milk is compatible with the farm water, the following steps are performed: (a) the actual α18m,t value is obtained using the measured values for milk and farm water by Equation 1; (b) the obtained α18m,t value is then compared with α^18t, which is estimated for time *t* using Equation 6b.

The two values are in good agreement when the following relationship is valid:

(8) αm,t18−α^18t≤uαm,t18−α^18t

where αm,t18−α^18t is the absolute difference between the measured and the calculated values. Given that the α and δ values are very close to unity, the following relationship holds for u:

uαm,t18−α^18t≈u2αm,t18+u2α^18t≈u2δO18/O16M,m,t+u2δO18/O16W,m,t+u2α^18t,

where uδO18/O16M,m,t = 0.15‰, uδO18/O16W,m,t = 0.08‰, and u(α^18t) = 0.42‰. With these numbers, the following value for u is obtained:

uαm,t18−α^18t=0.45‰.

For the four new cattle sheds, the difference αm,t18−α^18t is always < 0.45‰ (Table 2a). Thus, the two‐step verification model expressed by Equations 5 and 6b works well in the investigated region. Moreover, if the farm water is not available for stable isotope analysis and cannot be compared with milk water, it is necessary to have a database of the isotopic values of the groundwater of the Parmigiano‐Reggiano production area. For the Po Valley south of the Po River, there are many data in the literature and particularly in the work of Martinelli et al.^22^

In the next step we checked four samples, L1, L2, L3, and L4, declared by farmers to have originated from four locations of the area of production of Parmigiano‐Reggiano (Table 2b). First, the isotopic values for the milk water were considered to check if the milk is compatible with the region. As presented in Table 2b all four samples L1, L2, L3, and L4 are compatible with the area. Since the farm water was not available the isotope data from groundwater reported by Martinelli et al^22^ from declared farms were taken to calculate α18m,t. The α^18t value at time *t* is then estimated using Equation 6b and compared with α18m,t (Table 2b). Despite the fact that the values for the milk water agree with Equation 8, it is evident that, based on the *α* values, three samples are not compatible with their declared origin (marked italic and bold in Table 2b). It is possible that the origin declared by the farmers is suspicious.

## 5 CONCLUSIONS

This study has investigated the relationship between the stable isotopic signatures of farm water and milk water as a tool for verifying if milk originates from the Parmigiano‐Reggiano production region. It was shown that drinking water is the main factor influencing the isotope signature of the milk, but does not explain all of the observed variance. Other factors such as seasonal temperature variations that can induce a metabolic response in the animals can add to the observed isotopic variation in milk water. Thus, to indicate provenance, it is essential to analyze both milk and farm water. If this is not possible, the isotopic composition of groundwater from the investigated region can be used in its place. The study also found that the variation in δ(^18^O/^16^O)_W_ values in milk and the fractionation factor α18m,t is more dependent on sampling time rather than on the different cattle sheds. A 4^th^ order polynomial regression explains the dependence of the milk isotope features on the farm water isotopic composition. It is expected that the shape of the regression line depends on the variation in temperature between winter and summer and the different lifestyle of the cows (indoors or outdoors). Overall, the milk origin can be determined by two‐step verification using the following criteria: (i) the difference between the measured and calculated δ(^18^O/^16^O)_W_ values of milk using 4th order polynomial regression has to be ≤1.51‰, and (ii) the difference between the calculated and estimated milk–water fractionation factor has to be ≤0.45‰. Only if both conditions are valid can the milk be said to originate from the Parmigiano‐Reggiano production region. Clearly, such criteria will need to be verified by expanding this study over several years. Nevertheless, the proposed procedure may represent an additional tool for checking milk provenance, and, although it was developed for the Parmigiano‐Reggiano region, it can be easily tested and adopted for other regions of interest.

### PEER REVIEW

The peer review history for this article is available at https://publons.com/publon/10.1002/rcm.9160.

## Supporting information


**Table S1.** d(2H/1H), d(18O/16O), and related data for the investigated cattle sheds

## Data Availability

Data available in article supplementary material.
